# Antibody Response to Canine Parvovirus Vaccination in Dogs with Hypothyroidism Treated with Levothyroxine

**DOI:** 10.3390/vaccines9020180

**Published:** 2021-02-20

**Authors:** Michèle Bergmann, Monika Freisl, Katrin Hartmann, Stephanie Speck, Uwe Truyen, Yury Zablotski, Matthias Mayr, Astrid Wehner

**Affiliations:** 1 Clinic of Small Animal Medicine, LMU Munich, Veterinaerstrasse 13, 80539 Munich, Germany; m.freisl@medizinische-kleintierklinik.de (M.F.); hartmann@lmu.de (K.H.); Y.Zablotski@med.vetmed.uni-muenchen.de (Y.Z.); M.mayr@medizinische-kleintierklinik.de (M.M.); A.wehner@medizinische-kleintierklinik.de (A.W.); 2 Institute of Animal Hygiene and Veterinary Public Health, University of Leipzig, An den Tierkliniken 1, 04103 Leipzig, Germany; stephanie@speck-kaysser.de (S.S.); truyen@vetmed.uni-leipzig.de (U.T.)

**Keywords:** CPV, levothyroxine, T4, protection, immunosuppression, MLV, titer

## Abstract

(1) Background: No information is available on how dogs with hypothyroidism (HypoT) respond to vaccination. This study measured pre- and post-vaccination anti-canine parvovirus (CPV) antibodies in dogs with HypoT treated with levothyroxine and compared the results to those of healthy dogs. (2) Methods: Six dogs with HypoT and healthy age-matched control dogs (n = 23) were vaccinated against CPV with a modified-live vaccine. Hemagglutination inhibition was used to measure antibodies on days 0, 7, and 28. The comparison of the vaccination response of dogs with HypoT and healthy dogs were performed with univariate analysis. (3) Results: Pre-vaccination antibodies (≥10) were detected in 100% of dogs with HypoT (6/6; 95% CI: 55.7–100) and in 100% of healthy dogs (23/23; 95% CI: 83.1–100.0). A ≥4-fold titer increase was observed in none of the dogs with HypoT and in 4.3% of the healthy dogs (1/23; CI_95%_: <0.01–22.7). Mild vaccine-associated adverse events (VAAEs) were detected in 33.3% of the dogs with HypoT (2/6; 95% CI: 9.3–70.4) and in 43.5% (10/23; 95% CI: 25.6–63.2) of the healthy dogs. (4) Conclusions: There was neither a significant difference in the dogs’ pre-vaccination antibodies (*p* = 1.000), or vaccination response (*p* = 0.735), nor in the occurrence of post-vaccination VAAEs (*p* = 0.798). The vaccination response in dogs with levothyroxine-treated HypoT seems to be similar to that of healthy dogs.

## 1. Introduction

Canine parvovirus (CPV) is highly contagious and infection can be fatal if unprotected dogs are exposed to the virus [1]; thus, all dogs should be protected at any time [2]. Vaccination with this core component induces excellent immunity against infection at least in healthy dogs; nearly all of these dogs develop anti-CPV antibodies, indicating protection [2,3,4].

There is a complex relationship between the immune system and the neuroendocrine system. Several immune cells contain receptors for neuroendocrine hormones and recent evidence indicates that thyroid hormones, such as L-thyroxine (T4), maintain specific immune responses, including cell-mediated immunity, natural killer cell activity, antiviral action of interferons, as well as the proliferation of T and B lymphocytes [5]. Hypothyroidism (HypoT) is a common endocrinopathy in dogs [6]. Although its true prevalence remains largely unknown, many dogs are presented with or are treated for HypoT [7,8]. It is currently unknown whether these dogs develop and maintain (long-lasting) immunity by vaccination with modified live CPV.

So far, only a few experimental studies exist on the effect of thyroid hormones on the humoral immune response. In one of these studies, raising or lowering circulating thyroid hormones had no effect on the antibody response in domestic fowl [9]. Further studies evaluating antibody response in hypothyroid rodents are contradictory, showing either an enhanced [10,11] or a suppressed antibody response [12,13]. So far, there are no data in dogs or in humans.

Furthermore, it has been questioned whether the vaccination of dogs with HypoT is safe. It has been suggested that the common occurrence of HypoT in the dog population might be related to the increased use of modified live virus (MLV) vaccines and the induction of autoantibodies [14]; signs of HypoT could thus be triggered after vaccination even in dogs that are well-controlled at the time of vaccination [3]. Therefore, the aim of this study was to measure pre- and post-vaccination anti-CPV antibodies in dogs with HypoT treated with levothyroxine and compare the results to those for healthy dogs.

## 2. Materials and Methods

### 2.1. Study Population

Dogs with HypoT (n = 6) were patients of the Clinic of Small Animal Medicine, Centre for Clinical Veterinary Medicine, LMU Munich or a private practice in Southern Germany. Healthy dogs (n = 23) were presented for their annual vaccination to the same clinic or private practice or to a charity organisation. The study protocol was approved by the Government of Upper Bavaria, reference number 55.2–1–54–2532.3–61–11. Dogs were only included if they had received their last vaccine >12 and ≤15 months ago. Dogs that had received antibody preparations within the last 12 months were excluded.

Dogs with HypoT had to have a diagnosis of HypoT and the disease had to be well-controlled at the time of vaccination. Suspicion of HypoT was based on history, physical examination findings, and the results of laboratory testing (hematology and biochemistry profile) that are typically reported for dogs with HypoT [6,7,15,16]. A diagnosis of HypoT was confirmed if endogenous thyroid-stimulating hormone (TSH) was increased and T4 was below the reference range. If endogenous TSH was within the reference range, the diagnosis was based on a low T4 and free thyroxine (fT4) value and additionally the resolution of clinical signs with levothyroxine treatment. Dogs with an elevated TSH were classified as having primary HypoT. Dogs with a normal TSH were classified as having unclassified HypoT [15,17]. Dogs being fed with a homemade diet or bone and raw meat were excluded to avoid any influence of ingested thyroid tissue on their thyroid hormone levels [18]. Dogs from the HypoT group were examined for the presence of concurrent diseases. The control of HypoT was based on the resolution of clinical signs (e.g., lethargy, exercise intolerance, weight gain), physical examination findings (e.g., haircoat, general appearance), laboratory data (e.g., hypercholesterolemia, elevated liver enzymes, elevated fructosamine), and a T4 post-pill concentration that was within the reference range or slightly above and was classified as “good” or “moderate” control.

The group of healthy dogs was age-matched (≥4 years of age) and included only dogs that had (1) no history of illness, anesthesia, surgery, or medical treatment (besides deworming) during the last 4 weeks and (2) no remarkable findings in physical examination.

Information on the dogs’ signalment, origin, and environment, as well as data on the vaccination history (previous vaccinations, complete vaccination series, time since last vaccination) and medical history were collected.

### 2.2. Study Protocol

Physical examination was performed on days 0, 7, and 28 in order to determine the health status of all dogs. All the dogs received a single dose of a combined MLV vaccine containing CPV-2 strain 154 with a viral titer of 10^7.0–8.4^ TCID_50_ as well as canine distemper virus (CDV) and canine adenovirus-2 (CAV-2) (Nobivac^®^ SHP, MSD) on day 0. Owners had to pay special attention to the occurrence of vaccine-associated adverse events (VAAEs) or further abnormalities related to the dog´s health and behavior. Serum samples were taken on days 0, 7, and 28 for the evaluation of pre- and post-vaccination anti-CPV antibodies.

### 2.3. Detection of Antibodies by Hemagglutination Inhibition

For the detection of anti-CPV antibodies, serum samples were frozen at −20 °C and tested by hemagglutination inhibition (HI) at the end of the study with a protocol based on Carmichael and coworkers (1980) using 8 hemagglutinating units of CPV-2, strain vBI 265 (Provided by: James A. Baker Institute for Animal Health, College of Veterinary Medicine, Cornell University, 235 Hungerford Hill Road, Ithaca, NY 14853, USA) [19].

The highest dilution that completely inhibited the hemagglutination of CPV antigen was defined as the endpoint. The evaluation of HI was performed by 2 independent investigators blinded to the history of the patients; divergent results were rechecked by a third independent and blinded investigator.

Anti-CPV antibody titers ≥10 (with 10 being the first dilution) were considered positive. Dogs with an at least 4-fold titer increase (=2 titer steps) were considered as “responding to vaccination” [20]. Dogs that had no detectable anti-CPV antibodies before and after vaccination were defined as “non-responders”.

### 2.4. Statistical Analysis 

Data analysis was performed using R 4.0.3 (2020-10-10, R Foundation for Statistical Computing, Vienna, Austria). Basic non-parametric bootstrapping with 1000 resamples and replacement was used to calculate the means and 95% confidence intervals for the numeric variables age, bodyweight, and time since last vaccination of dogs with HypoT and healthy dogs. Bayesian logistic regression was used to determine significant differences in the age, body weight, and time since last vaccination between dogs from the 2 respective groups; due to the non-normally distributed and heteroscedastic characteristics, the variable time since last vaccination was logarithmized. In order to verify the modelling results, differences were additionally studied using classic statistical tests. Therefore, the normality of data distribution was assessed with the Shapiro–Wilk normality test and visually using Quantile-Quantile plots. Non-normally distributed data were further analyzed using a non-parametric 2 sample Mann–Whitney test, while normally distributed data were further checked for the homogeneity of variance across groups via the Bartlett test. Normally distributed and homogeneous data were assessed by the Student’s *t*-test; normally distributed non-homogeneous data were assessed by Welsh’s *t*-test. Bayesian logistic regression was also used to compare (1) the presence of anti-CPV antibodies before vaccination between dogs with HypoT and healthy dogs, (2) anti-CPV antibody response after vaccination, and (3) the occurrence of VAAEs. The normality and homoscedasticity of residuals of all Bayesian models were assessed via visual residual-diagnostics. Results with a *p*-value < 0.05 were considered statistically significant; results with a *p*-values between 0.1 and 0.05 were considered suggestive.

## 3. Results

### 3.1. Dog Population

The present study included 6 dogs with HypoT (Table 1). Of these dogs, 4 dogs were male (66.7%) and 2 dogs were female (33.3%). Their ages ranged between 7 and 13 years (mean age: 9.32 years; 95%CI: 7.97–11.2). Body weight ranged between 26 and 46 kg (mean weight: 37.2; 95%CI: 32.3–42.1). Three dogs lived in an urban and 3 dogs in a rural area (each 50%). Two dogs had >5 daily contacts with other dogs, 2 dogs had 3–5 daily contacts (33.3%), and 2 dogs had ≤2 daily contacts with other dogs (each 33.3%). Primary HypoT was diagnosed in 4 dogs (66.7%). Unclassified HypoT was diagnosed in 2 dogs (33.3%). In all dogs, HypoT was well controlled during the whole study course. The median dose of levothyroxine (Forthyron^®^, Dechra, Aulendorf, Germany) was 9.4 mg/kg twice daily (BID) (range: 7.7–11 mg/kg). Treatment with levothyroxine had been started immediately after the diagnosis of HypoT in all dogs. The median time between establishment of HypoT diagnosis and vaccination (on study day 0) was 997 days (range: 253–2492 days). Two dogs had concurrent diseases. One dog suffered from symmetrical lupoid onychodystrophy, which was well-controlled. At the time of the study entry, increased liver enzymes were noted in another dog which had been treated for mitral valve disease and bradyarrhythmia before. During the course of the study, this dog was diagnosed with lymphoma and additional medications were given (Table 1).

Nineteen of the 23 dogs in the healthy group were female (82.6%) and 4 dogs were male (17.4%). Age ranged between 4 to 13 years (mean age: 7.37 years; 95%CI: 6.44–8.42). Body weight ranged between 17 to 26 kg (mean weight: 21.7; 95%CI: 17.2–26.7); 15 dogs were neutered (65.2%) and 8 dogs were intact (34.8%). Twelve dogs came from urban areas (52.2%) and 11 dogs from rural areas (47.8%). Two dogs had >5 daily contacts with other dogs (8.7%), 17 dogs had 3–5 daily contacts (73.9%), and 4 dogs had ≤2 daily contacts with other dogs (17.4%).

All dogs with HypoT and all healthy dogs had been vaccinated in the past (>12 and ≤15 months ago). None of the dogs with HypoT and only 7 of the dogs in the healthy group (30.4%) had received a primary immunization series according to expert guidelines [2,3]. Dogs were considered to have received a full primary vaccination if they had received CPV vaccinations in 3–4 weeks intervals and the last vaccination with at least 14–16 weeks of age, followed by a booster after 11–13 months. In dogs ≥12 weeks, immunization was considered complete if they had received 2 vaccinations every 3–4 weeks and a booster after 11–13 months. After the primary immunization series, subsequent boosters had to be given at least every 3 years [2,3]. The mean time since the last vaccination was 1.04 years (95% CI: 1.02–1.08) in dogs with HypoT and 1.06 years (95% CI: 1.04–1.08) in healthy dogs.

There was no significant difference between the mean age of the dogs with HypoT (9.3 years) and healthy dogs (7.4 years) (Bayesian *p*-value = 0.091; student´s *p*-value = 0.089). There was also no significant difference between the median time since last vaccination of dogs with HypoT (1.03 years) and healthy dogs (1.06 years) (Bayesian *p*-value = 0.540; Mann–Whitney *p*-value = 0.552). The mean body weight of the dogs with HypoT (37.2 kg) and healthy dogs (21.7 kg) differed significantly (Bayesian *p*-value = 0.005; student´s *p*-value = 0.005).

### 3.2. Response to Vaccination 

The vaccination response of all dogs is shown in Table 2 and Table 3. All dogs with HypoT (100%; 6/6, 95% CI: 55.7–100) had anti-CPV antibodies (≥10) before vaccination on day 0 (median antibody titer: 160; range: 80–2560) (Table 1). None of these dogs had a ≥4-fold increase after vaccination (median antibody titer on day 7 and 28: each 240; range on day 7 and 28: each 80–2560). VAAEs (mild gastrointestinal signs) were observed in 2/6 of the dogs with HypoT (33.3%; 95% CI: 9.3–70.4) by the owners; one of the 2 dogs also had a slightly reduced general condition with less activity for a few days after vaccination.

All healthy dogs, 23/23 (100%; 95% CI: 83.1–100.0) had pre-vaccination antibodies ≥10 (median antibody titer: 160; range: 40–1280). Response to vaccination was observed in 1/23 (4.3%; 95% CI: <0.01–22.8) of the healthy dogs (median antibody titer on day 7 and day 28: each 320; range on day 7: 40–5210; range on day 28: 20–2560). In 10/23 (43.5%; 95% CI: 25.6–63.2) of the healthy dogs, VAAEs were described by the owners and included a slightly reduced general condition with less activity (n = 7) or mild gastrointestinal signs (n = 4) for a few days after vaccination; 1 dog showed both a transiently reduced general condition and gastrointestinal signs.

### 3.3. Comparison of Dogs with HypoT and Healthy Dogs

No significant difference in the presence of pre-vaccination antibody titers ≥10 on day 0 (*p* = 1.000) could be found between dogs with HypoT and healthy dogs. In addition, there was no significant difference in the response to vaccination between dogs with HypoT and healthy dogs (*p* = 0.735). Additionally, no difference in the occurrence of VAAEs (*p* = 0.798) was detected.

## 4. Discussion

All the dogs with HypoT in the present study had anti-CPV antibodies before vaccination, suggesting that they had effectively reacted to previous vaccinations (or infection) and were protected against parvovirosis. It has been proposed that altering thyroid hormones could have an influence on the maintenance of the immune function and thus on the vaccination response [21]. Several immune cells contain receptors for thyroid hormones, indicating relationships between thyroid hormones and the immune system. In human medicine, thyroid hormones in higher concentrations but still within normal physiological ranges were shown to be positively associated with immune function—e.g., with the proliferation of monocytes and several lymphocyte subpopulations [5]—leading to a greater responsiveness of the immune system. Furthermore, thyroid hormones can affect endogenous glucocorticoid levels [22,23,24]. The administration of triiodothyronine (T3) and T4 suppressed the basal and ACTH-stimulated levels of blood cortisol, at least in rats [23]; in contrast, low levels of thyroid hormones could lead to a chronic elevation of endogenous blood cortisol and thus impaired immune function [22], although a previous study revealed that the immune response of dogs with treated hyperadrenocorticism (HAC) to MLV vaccination against CPV was not significantly impaired in comparison to that of healthy dogs [25].

This is the first study that examines and compares the response of modified life CPV vaccination in dogs with HypoT to that of healthy ones. The findings of the present study are especially important for implementing vaccination guidelines in dogs with HypoT; in addition, the results could serve as a model for vaccination in humans with HypoT.

Functional canine HypoT is mainly caused by primary diseases of the thyroid gland [26]. Lymphocytic thyroiditis is the leading cause affecting more than 50% of cases [27,28,29,30]. In humans, Hashimoto thyroiditis, a chronic inflammation of the thyroid gland, represents the most common cause of hypothyroidism; it is also considered to be autoimmune in origin. In dogs as well as in humans, it is currently unknown whether HypoT leads to (long-lasting) immunity after vaccination with modified live vaccines. 

An adequate vaccination response (≥4-fold titer increase) could not be observed in any of the dogs in the HypoT group; only one dog developed a titer increase (but only one titer step, which is considered to be negligible). Even so, most of the healthy dogs (95.7%) did not develop an adequate vaccination response either. The most likely cause for not having a ≥4-fold titer increase in both groups is pre-existing antibodies. A previous study has already demonstrated that healthy dogs with pre-existing anti-CPV antibody titers ≥80 are more likely to lack vaccination response than dogs with titers <80 [20], since pre-existing neutralizing antibodies can bind to vaccine virus and thus prevent an active immune response. This is why regular CPV re-vaccinations are not advised in adult dogs with pre-existing antibodies. 

It is presently uncertain whether vaccination in dogs with HypoT is safe. Similar to autoimmune thyroiditis in humans, a primarily immune-mediated disease is suspected in dogs with HypoT [26,31] or at least a process with defective immune regulation [30,32]. Although the destruction of canine thyroid tissue is largely explained by direct T-cell toxicity, autoantibodies are also thought to be important in the pathogenesis of canine HypoT [33,34]. Due to the high frequency of lymphocytic thyroiditis in dogs and the frequent use of vaccines in veterinary medicine (at least in the past), it has been suggested that lymphocytic thyroiditis could be related to or triggered by a type II hypersensitivity reaction following the overstimulation of the immune system by vaccines, leading to the induction of autoantibodies [14,35,36,37]. In humans, autoantibodies against thyroid antigens (thyroperoxidase and thyroglobulin) are usually present, although the role of those antibodies in the disease process still remains unclear [38,39]. A comparable diagnostic approach has been made in hypothyroid dogs, in which, in addition to thyroglobulin autoantibodies (TgAA) [32,40,41,42] antibodies against T3 [40,41,43,44] and T4 [41,44] could also be found. In contrast, antibodies against thyroid peroxidase have not been detected [14,45]. HogenEsch and colleagues were able to demonstrate that various autoantibodies were induced when dogs were vaccinated according to a standard vaccination protocol. However, an increase in thyroglobulin antibodies could not be detected in the vaccinated dogs, all of them beagles. Furthermore, no clear indication of thyroid dysfunction could be detected. A “nodule” was found in the thyroid gland in only one dog in that study [14]. According to the authors, this is known as a common lesion in beagles [46] and could be interpreted as an early manifestation of thyroiditis [14]. 

Besides the induction of autoantibodies due to hypersensitivity, it is also conceivable that the contamination of vaccines with foreign thyroglobulin, primarily of bovine serum, could induce thyroglobulin antibodies in vaccinated dogs [4]. Bovine serum is commonly used in the production of cell culture-based vaccines, especially in high-titer CPV (and CDV) vaccines [47,48,49]. It could therefore be assumed that HypoT and subsequent clinical disease could be triggered after vaccination in dogs predisposed to HypoT or that signs of HypoT reoccur in dogs with HypoT that were actually well-controlled at the time of vaccination [3,50]. Scott-Moncrieff and colleagues targeted canine and bovine thyroglobulin antibodies in laboratory beagles (n = 20) as well as privately owned adult dogs (n = 16) at defined times before and after vaccination. Post-mortem histopathological examinations of the Beagle at the age of 5.5 years found no evidence that repeated routine vaccinations lead to immune-mediated thyroiditis in dogs. However, since an unexpectedly high prevalence of thyroiditis also occurred in the unvaccinated control group, the scope of the investigation for the detection of such an association was limited [51].

All the dogs from the HypoT group in the present study were well-controlled and signs of HypoT had not reoccurred after vaccination during the study course. However, since cell destruction can be caused by different things (e.g., by the complement system, phagocytosis, or natural killer cells), clinical consequences can occur at different time points; destruction via macrophages or natural killer cells can take days to weeks, whereas cytolysis via the complement systems is much faster [52]. Taking a possible combination of predisposing factors for development of immune-mediated HypoT into account, antibody testing against important infectious diseases should be recommended particularly in dogs with HypoT, and regular re-vaccinations should only be considered when antibodies cannot be detected.

Two of the dogs in the HypoT group in the present study had concurrent diseases. One dog was presented with newly diagnosed lymphoma and treated with prednisolone during the study course at an anti-inflammatory dose. In dogs, lymphoma can lead to reduced T-cell numbers [53] and even to changes in antibody production, especially when the tumor cells secrete paraproteins—i.e., abnormal immunoglobulins—which simultaneously interfere with normal antibody production [54,55]. Although a human medicine meta-analysis revealed that tumor patients develop an impaired humoral immune response to vaccinations before tumor therapy [56], the immune competence of the dog with lymphoma might have been additionally impaired due to the glucocorticoid treatment, as the suppression of pituitary and adrenal function was noted in dogs that were treated for 35 days with anti-inflammatory doses of prednisone [57]. However, the vaccination response of the dog with lymphoma in the present study did not differ to that of healthy dogs, and the dog showed no VAAEs. The second dog from the HypoT group had concurrent symmetrical lupoid onychodystrophy (SLO), which has also been hypothesized to be activated by repeated vaccinations [48]. That dog was well-controlled with oral pentoxifylline and vitamin E, and signs of SLO did not worsen after vaccination; however, the dog developed mild gastrointestinal signs for a few days after vaccination.

To date, there are no data on whether individuals with HypoT are more likely to develop VAAEs after MLV vaccination. Lethargy and gastrointestinal signs after vaccination were commonly observed in the present study and can result from the owners´ special attention toward VAAEs. The occurrence of VAAEs did not differ significantly between dogs from the HypoT group and healthy dogs. Only one third of the dogs in the HypoT group showed mild gastrointestinal signs. Since no other signs of HypoT were reported in the dogs after vaccination and due to the mild and self-limiting nature of the gastrointestinal signs, they presumably resulted from active CPV replication in the gastrointestinal cells and not from the worsening of HypoT. However, an increased replication of MLVs in dogs with HypoT might occur due to a declined function of the innate immune system (e.g., monocytes), which is a first-line defense mechanism against viral infections [58]. Data from the present study suggest that MLVs can therefore be considered safe for dogs with HypoT, at least when they are well-controlled. 

In 4 dogs, primary HypoT was diagnosed based on increased TSH and low T4 concentrations in addition to the presence of clinical signs. In 2 dogs, the origin of HypoT could not be determined, as their endogenous TSH concentrations were low. Their diagnosis was based on low T4 and free thyroxine (fT4) concentrations and the presence of clinical signs that were resolved with levothyroxine treatment. In dogs, HypoT is the consequence of primary disease in almost all cases. However, in up to 40% of cases increased TSH levels are not present [59]. It has been demonstrated that TSH can decrease over time in dogs with surgical induced hypothyroidism as a consequence of vacuolar changes of thyrotrophic cells of the adenohypophysis [60].

The prevalence of thyroid function decreases with age in both humans and dogs [61,62]. Concerning the age distribution, lymphocytic thyroiditis is rarely described in dogs younger than 2 years and reaches its highest peak at 4–5 years, whereas idiopathic atrophy (or TgAA-negative HypoT) affects dogs from 4 years onwards and peaks around 8–9 years [6]. Therefore, only dogs ≥4 years were included in the present study.

The main limitation of the study was the low number of dogs in the HypoT group, making the assessment of the vaccination response difficult. Long-term studies involving larger numbers of dogs with HypoT and different stages of disease and/or its medical control would be useful. Furthermore, the investigation of cellular immunity would be desirable for future research. 

## 5. Conclusions

All the dogs with HypoT had pre-vaccination antibodies against CPV indicating protection. Vaccination response in dogs with well-controlled HypoT was similar to that of healthy dogs, and thus the immune function seems to be comparable. However, mild VAAEs were commonly observed after vaccination. Thus, measurement of antibodies against CPV infection would therefore be an excellent possibility in dogs with HypoT to confirm that protection is present instead of routine re-vaccination. Long-term studies involving larger numbers of dogs with HypoT and different stages of disease are needed.

## Figures and Tables

**Table 1:** Signalment, history, and pre-vaccination antibody titer against canine parvovirus (CPV) of the dogs with hypothyroidism (HypoT) treated with levothyroxine and their response to vaccination.

Dog	Signalment, Weight	Origin of HypoT ^a^	Diagnosis Based on	TSH ^b^ and Thyroid Hormones at Diagnosis (Reference Range)	Time Since Diagnosis (Days)	Thyroid Medication	Thyroid Control at Study Start (Reference Range)	Concurrent Disease (Treatment)	Time Since Last Vaccination (Years)	CPV ^c^ Antibody Titer on Day 0, 7, 28			VAAEs ^d^
1	Golden Retriever, 8 years, female spayed, 41 kg ^e^	primary HypoT	clinical signs and TSH↑ and T4 ^f^↓	TSH 2.71 ng ^g^/mL ^h^ (<0.5), T4↓ 1.1 µg ^i^/dl ^j^ (1.5–4)	297	Forthyron^®^ 400 µg BID ^k^	good control T4 4.4 µg/dl (1–4)	none	1.1	160	320	320	none
2	Border Collie, 13 years, male neutered, 26 kg	primary HypoT	clinical signs and TSH↑ and T4↓	TSH 8.59 ng/mL (<0.4), T4 0.74 µg/dl (1.5–4.5)	521	Forthyron^®^ 200 µg BID	good control T4 2.38 µg/dl (1.5–4.5)	mitral valve disease and bradyarrhythmia (pimobendane); lymphoma (prednisolone, silymarin, metamizol)	1.0	80	80	80	none
3	Hovawart, 9 years, male neutered, 38 kg	primary HypoT	clinical signs and TSH↑ and T4↓	TSH 8.03 (<0.5), T4 0.6 µg/dl (1.5–4.5)	2492	Forthyron^®^ 200 µg BID	good control T4 1.9 µg/dl (1–4)	none	1.0	160	160	160	none
4	Rhodesian Ridgeback 7 years, male, 46 kg	primary HypoT	clinical signs and TSH↑ and T4↓	TSH 1.69 ng/mL (<0.5), T4 0.5 µg/dl (1–4)	1162	Forthyron^®^ 400 µg BID	good control T4 3.4 µg/dl (1–4)	symmetrical lupoid onychodystrophy (Vitamin E, pentoxifyllin), mitral valve disease compensated	1.3	2560	2560	1280	GI ^l^ signs on days 0–7 after vaccination
5	Mix breed, 9 years, female spayed, 34 kg	unclassified HypoT	clinical signs, T4↓ and fT4 ^m^↓, positive response to replacement therapy	TSH 0.26 ng/mL (0.02–0.4), T4 < 0.5 µg/dl (1.5–4.5), fT4 0.57 ng/dl (0.6–3.7)	253	Forthyron^®^ 300 µg BID	good control T4 4.7 µg/dl (1–4)	none	1.0	2560	2560	2560	GI signs, lethargy on days 0–7 after vaccination
6	Golden Retriever, 7 years, male neutered, 37 kg	unclassified HypoT	clinical signs, T4↓ and fT4↓, positive response to replacement therapy	TSH 0.22 ng/mL (0.02–0.4), T4 0.72 µg/dl (1.5–4.5), fT4 0.41 ng/dl (0.6–3.7)	1256	Forthyron^®^ 200 µg BID	good control T4 2.17 µg/dl (1–4)	none	1.0	80	80	80	none

^a^ HypoT = hypothyroidism; ^b^ TSH = thyroid-stimulating hormone; ^c^ CPV = canine parvovirus; ^d^ VAAEs = vaccine-associated adverse events; ^e^ kg = kilogram; ^f^ T4 = thyroxine; ^g^ ng = nanogram; ^h^ mL = milliliter; ^i^ µg = microgram; ^j^ dl = deciliter; ^k^ BID = twice daily; ^l^ GI = gastrointestinal; ^m^ fT4 = free thyroxine.

**Table 2:** Comparison of the humoral immune response of dogs with hypothyroidism (HypoT) treated with thyroxine and healthy dogs after modified live virus vaccination against canine parvovirus using Bayesian logistic regression.

		Total	Dogs with HypoT	Healthy Dogs	*p* ^a^
total			6	23	
pre-vaccination antibody titer (n ^b^ = 29)	<10	0	0/6 (0.0%)	0/23 (0.0%)	1.000
	≥10	29	6/6 (100.0%)	23/23 (100.0%)	
≥4-fold titer increase (n = 29)	yes	1	0/6 (0.0%)	1/23 (4.3%)	0.735
	no	28	6/6 (100.0%)	22/23 (95.7%)	
vaccine-associated adverse events ^c^	yes	12	2/6 (33.3%)	10/23 (43.5%)	0.798
	no	17	4/6 (66.6%)	13/23 (56.5%)	

^a^*p* = *p*-value; ^b^ n = number of dogs; ^c^ based on owner reports and veterinary examination on days 7 and 28.

**Table 3:** Anti-canine parvovirus pre-vaccination antibody titer on day 0 and number of dogs with an ≥4-fold titer increase during the course of the study.

	Number of Dogs with A ≥4-fold Antibody Titer Increase with the Respective Pre-Vaccination Antibody Titer on Day 0	
Pre-Vaccination CPV ^a^ Antibody Titer on Day 0	Dogs with HypoT ^b^ (%)	Healthy Dogs (%)
<10	0/0 (0.0)	0/0 (0.0)
10	0/0 (0.0)	0/0 (0.0)
20	0/0 (0.0)	0/0 (0.0)
40	0/0 (0.0)	0/1 (0.0)
80	0/3 (0.0)	0/4 (0.0)
160	0/2 (0.0)	1/8 (12.5)
320	0/1 (0.0)	0/5 (0.0)
640	0/0 (0.0)	0/2 (0.0)
1280	0/0 (0.0)	0/3 (0.0)
Total number of dogs with ≥4-fold antibody titer increase	0/6 (0.0)	1/23 (4.3)

^a^ CPV = canine parvovirus, ^b^ HypoT = hypothyroidism.

## Data Availability

The authors confirm that the datasets analyzed during the study are available from the first author or the corresponding author upon reasonable request.
